# Evaluation of the North West London Diabetes Foot Care Transformation Project: A Mixed-Methods Evaluation

**DOI:** 10.5334/ijic.5956

**Published:** 2022-04-05

**Authors:** Raffaele Palladino, Ash More, Geva Greenfield, Nana Anokye, Elizabeth Pigott, Tony Willis, Gregg Edward, Azeem Majeed, Wing May Kong

**Affiliations:** 1 Department of Primary Care and Public Health, Imperial College School of Public Health, Imperial College, London, the UK; 2 Department of Public Health, University “Federico II” of Naples, Naples, Italy; 3 North West London Health and Care Partnership, NHS, London, the UK; 4 GP, Richford Gate Medical Practice, London, the UK; 5 Department of Epidemiology and Biostatistics, School of Public Health, Imperial College London, London, the UK; 6 MRC Centre for Environment and Health, Imperial College London, London, the UK; 7 Department of Diabetes and Endocrinology, Central Middlesex Hospital, LMWUH NHS Trust, London, the UK

**Keywords:** diabetes foot care, mixed-method evaluation, diabetes, protocol

## Abstract

**Introduction:**

Diabetes foot ulceration (DFU) presents an enormous burden to those living with diabetes and to the local health systems and economies. There is an increasing interest in implementing integrated care models to enhance the quality of care for people living with diabetes and related complications and the value of co-production approaches to achieve sustainable change. This paper aims to describe the evaluation methodology for the North West London (NWL) Diabetes Foot Care Transformation project.

**Description:**

A mixed methods design including: i) a quasi-experimental quantitative analysis assessing the impact of the implementation of the local secondary care multi-disciplinary diabetes foot team clinics on service utilisation and clinical outcomes (amputations and number of healed patients); ii) a phenomenological, qualitative study to explore patient and staff experience; and iii) a within-trial cost-effectiveness analysis (pre and post 2017) to evaluate the programme cost-effectiveness.

**Discussion and Conclusion:**

Demonstrating the impact of multidisciplinary, integrated care models and the value of co-production approaches is important for health providers and commissioners trying to improve health outcome. Evaluation is also needed to identify strategies to overcome barriers which might have reduced the impact of the programme and key elements for improvement.

## Introduction

Diabetes foot ulceration (DFU) presents an enormous burden to those living with diabetes and to local health economies with an estimated prevalence of 2.5% in people with diabetes [1]. DFU is associated with 5-year lower limb amputation rates of up to 20% [2] (approximately 7000 amputations annually in England alone) [1] and 5-year survival rates of less than 60% [2], lower than breast or prostate cancer. DFU accounts for 86% of inpatient costs for people with diabetes (£322 million in 2014/15 in England and Wales) [3]. The cost of DFU to the NHS is an estimated £1 billion per year [4].

The implementation of integrated care models to improve the outcomes of people with diabetes is becoming increasingly common in many countries [5,6,7]. Integrated care models strengthen people-centred health systems through the promotion of the comprehensive delivery of quality services across the life course [8]. Integrated care models for diabetes and diabetes complications care have the potential to improve patient outcomes, promote patient safety, increase patient satisfaction and optimise the use of resources [6]. Improvements in DFU care could prevent 80% of amputations [9]. The Multi-disciplinary Diabetes Foot Team (MDFT) is a multidisciplinary, integrated approach that improves DFU outcomes. In the UK, the National Institute for Clinical Excellence (NICE) recommends MDFT review within 24 hours for acute DFU (NICE NG 19) [10,11,12]. Evidence from the UK National Diabetes Footcare Audit [13] found integration of community foot protection teams and secondary care MDFT services and ease of pathway navigation were associated with improved foot outcomes.

**Figure 1:**
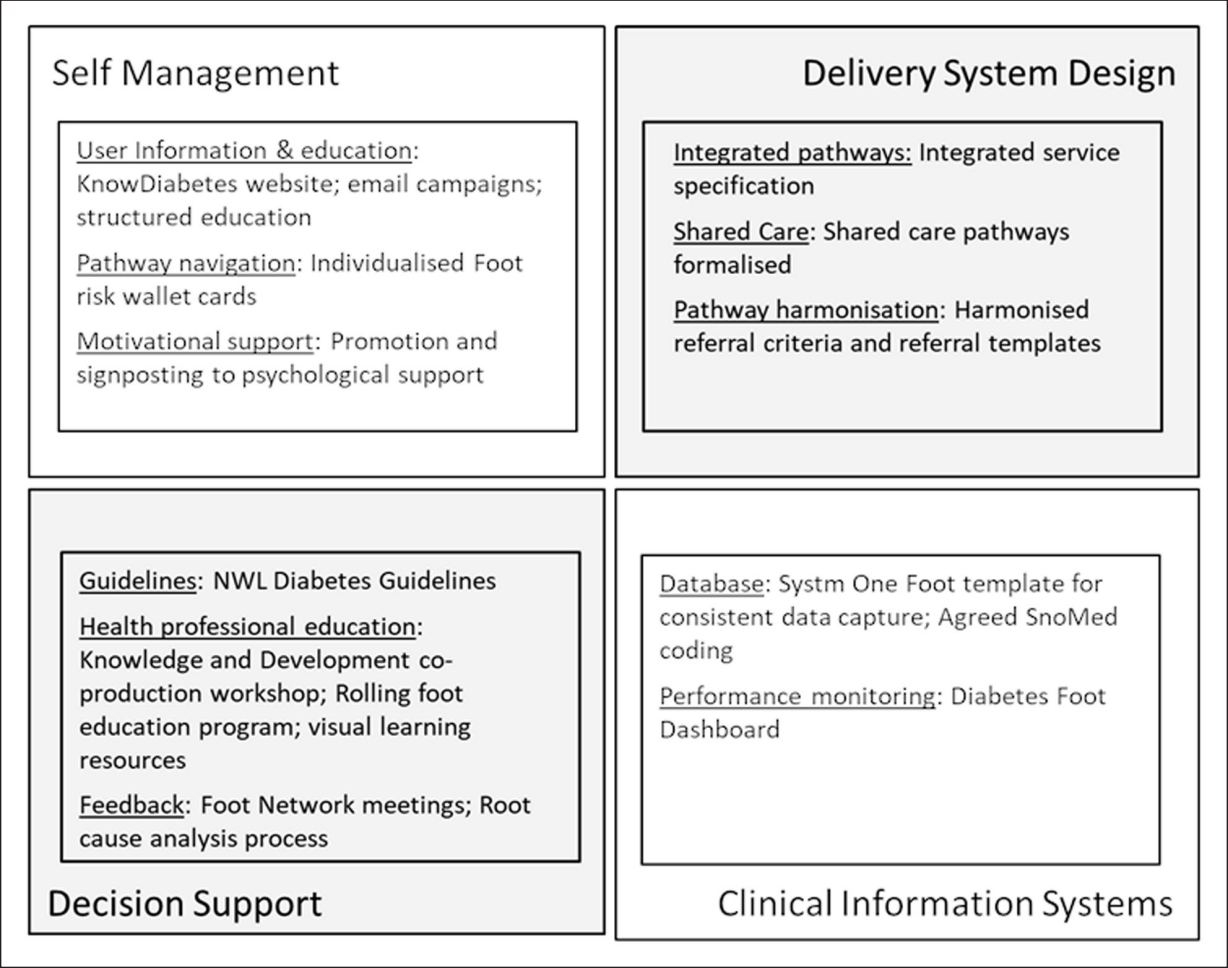
Overview of NWL MDFT Transformation project in terms of interventions in relation to Busetto’s model of integrated care for long term conditions.

In 2017 the NWL (North–West London) Diabetes Footcare Transformation project was launched as part of a wider NWL diabetes transformation program. Despite well-established MDFTs across NWL, quantitative analyses showed that there was significant variation in diabetes foot outcomes. The gap analysis suggested that better integration of care was needed. The project was designed according to the four key components of Integrated Care for long term conditions described by Busetto et al. [14]: i) Self-management through user information and education, pathway navigation, and motivational support; ii) Delivery System Design through specification of integrated pathways, formalisation of shared care, and pathway harmonisation; iii) Decision support for health providers through guidelines, health professional education, and feedback; and iv) Clinical information systems, through building a specific database and performance monitoring dashboard (***Figure 1***). ***Table 1*** summarises the project’s objectives.

**Table 1:** NWL MDFT objectives and associated goals.


PROJECT OBJECTIVES	ASSOCIATED GOALS

Reduce the rate for diabetic foot amputation in NWL	50% reduction in amputation rates by 2021

Improve patient care pathways by increasing referral rates and foot checks, reducing time from referral to presentation	Integrated pathways across primary, community and acute care services

Reduce unscheduled hospital admissions for diabetic foot and the length of stay	Reduction of the unscheduled hospital admissions

	Reduction of the length of stay by 1.5 days

Reduce inequalities in access to care and related health outcomes	Equitable service provision to ensure areas of greatest need are adequately resourced

Improve expertise, awareness, and confidence in managing diabetes foot complications among service users	Improve staff expertise via training on identification of foot emergencies

	Cultural change amongst key stakeholders regarding knowledge and importance of diabetes foot problems and commitment to sustainable quality improvement


The four areas for intervention were 1) Development of a Diabetes Foot Dashboard; 2) Harmonisation of pathways across NWL; 3) User engagement and 4) workforce development.

A co-production approach was used, creating a NWL Diabetes Foot Network of service users, foot teams, other health providers and commissioners (Appendix Figure 1) to design and implement an integrated care model for DFU across the 8 NWL Clinical Commissioning Groups, comprising a population of almost 150,000 people with diabetes (Appendix Table 1). At the inaugural Foot Network meeting priorities and actions for each of these intervention areas were produced. Specific actions were taken forward by a small, multi-stakeholder task and finish group (the NWL Diabetes Foot project group) and further refinement of interventions (e.g. creation of a NWL diabetes footcare service specification, design of visual training resources for health care professionals, development of service user facing digital resources for the NWL KnowDiabetes website [15]) carried out in the 4-monthly NWL Foot Network meetings. Six diabetes specialist podiatrists were recruited to form a new NWL MDFT to work across all acute and community sites in NWL and support the implementation of the project through job plans that crossed organisational boundaries, Support Foot Project groups and Network meetings and leading health professional training in diabetes footcare across acute and primary care sites.

Effective, integrated diabetes foot care involves multiple stakeholders. Co-production approaches support user centred solutions which are likely to lead to sustainable change and are being used increasingly in healthcare quality improvement [16]. However, co-production is also time consuming and robust evaluation of co-production methods is needed [16]. To the authors’ knowledge, robust methodologies for the evaluation of such a complex range of interventions developed using co-production for the care of diabetes complications have not been used before. This paper aims to describe the evaluation methodology for the NWL Diabetes Foot Care Transformation project. Central in developing this evaluation methodology was recognising the complexity of the multidisciplinary intervention in clinical, financial, strategic, and political contexts. In particular, due to the complexity in its implementation and challenges associated with the care of foot disease, this methodology was considered within the broader theory of complex intervention evaluation and also drawing from the UK Medical Research Council’s guidance [17].

## The Evaluation Framework

**Figure 2:**
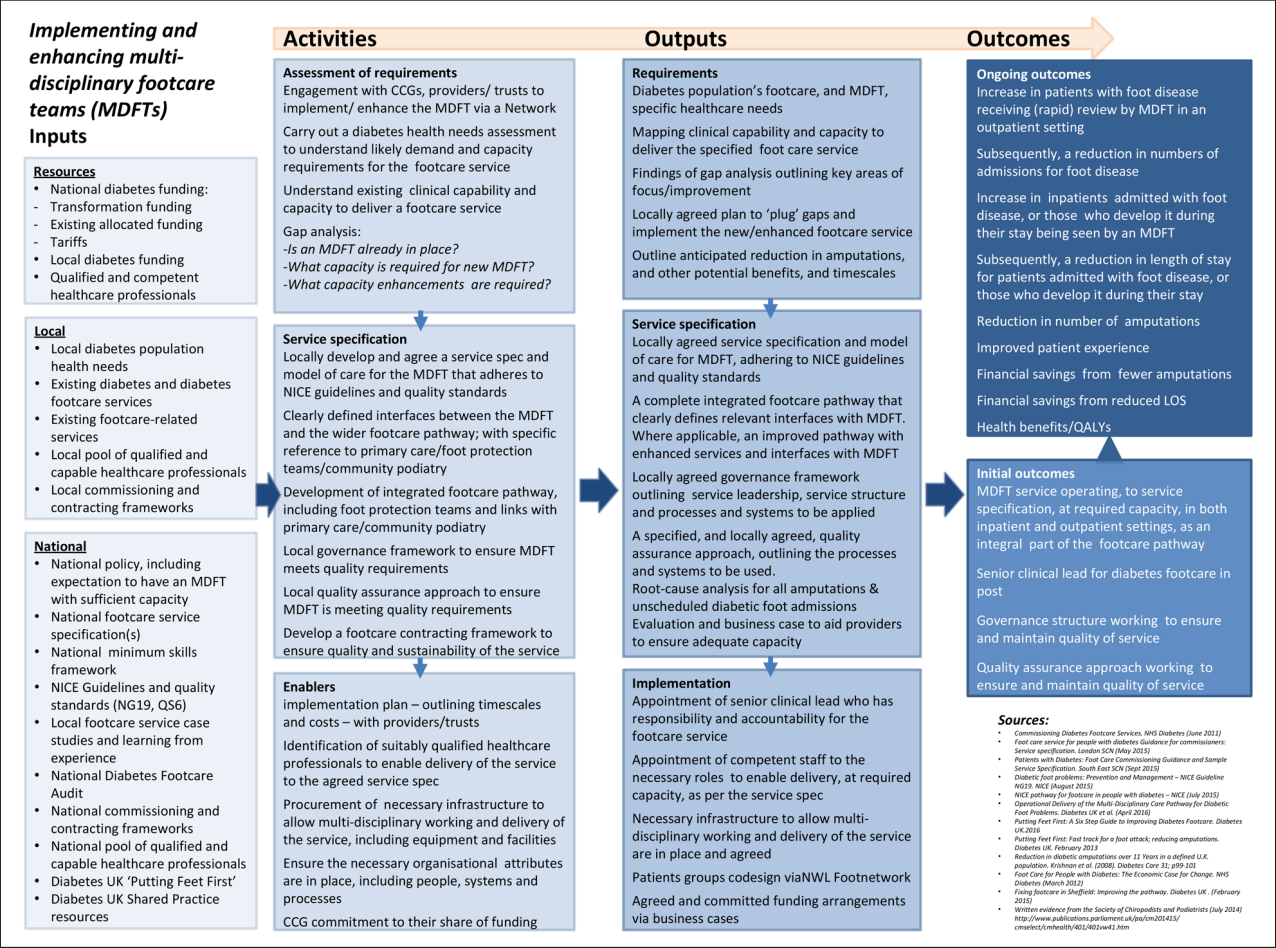
Logic model summarising the NWL MDFT Foot project.

The NWL Diabetes Foot Care Transformation project is a complex intervention whose framework is summarised in ***Figure 2***, a logic model including the shared relationships among the inputs, resources, activities, outputs, and outcomes for the project. To evaluate the project, a mixed-method approach was used made up of three different work streams: Work Stream 1 – Impact on service utilisation and clinical outcomes; Work Stream 2 – Patient and Staff Experience; Work Stream 3 – Cost-effectiveness.

### Work Stream 1 – Impact on service utilisation and clinical outcomes

The first work stream includes a quantitative quasi-experimental study aiming to assess the impact of the local MDFT service implementation within the Diabetes Foot Care Transformation project on service utilisation and clinical outcomes using a combination of data collected by the MDFT service and the Whole Systems Integrated Care (WSIC) dataset. The majority of people with diabetes foot complications will have neuropathy or arterial disease but no acute problem. They will be seen by the community foot protection team whose role is to prevent acute foot problems and rapidly escalate people when acute problems arise. The MDFT sees patients with complex diabetes foot complications. The majority of these (>95%) will be complex diabetes foot ulcers (e.g. chronic ulcers, moderate and severe infected ulcers including diabetes foot osteomyelitis and ischaemic ulcers).

#### Data sources

##### Multi-disciplinary Diabetes Foot Team service data

Information about MDFT inpatient and outpatient activity clinics has been recorded in a dedicated database. Collected data includes patients’ demographic characteristics, hospital site, date, and characteristics of the intervention, including diagnosis, treatment (routine treatment, dressing, ulcer debridement, vascular, neurological, or diabetic foot screen), and outcome (referral to Tier 3 – community foot services, Tier 4 – hospital services, discharge, other e.g. primary care).

##### The Northwest London Whole Systems Integrated Care dataset

The WSIC is one of the largest data sets in the UK, and comprises of linked coded data from primary care, secondary care, community, mental health and social care based in the NWL area [18]. Most of the 372 GP practices in NWL have subscribed to WSIC which contains the patient pathways and records of over 2.2 million patients in the area [19]. A NWL Foot Dashboard is in development which will draw data from the WSIC dataset to present key statistics related to diabetic foot patients across all pathways of diabetes footcare in NWL to support clinicians and commissioners.

##### Data analysis

In line with previous work evaluating similar interventions, for the evaluation of the NWL Diabetes Foot Care Transformation project the following process indicators will be selected

- Number of referrals to MDFT clinics
- Number of emergency admissions for foot disease
- Number of amputations
- Time to presentation
- Severity at presentation
- Number of healed patients (Data source: National Diabetes Foot Audit)

Collected data from the MDFT database will be monthly and yearly averaged. First access to the MDFT clinic will be considered as baseline for each individual. Descriptive statistics at baseline year on access to MDFT clinics for foot disease and clinical characteristics of the referred patients will be stratified by age, sex, and year. To assess unadjusted differences between groups identified within the set of all participants that are referred, univariate statistics including Chi-square, t-test, analysis of variance, and Kruskal-Wallis test will be employed, as appropriate. Multivariate mixed-effect generalised Poisson regression model will be employed to model change in trends over the study period, including referrals to MDFT clinics, emergency admissions for foot disease, number of healed patients. NWL population size will be included as offset in the regression analysis. Multivariate mixed-effect linear regression models will be employed to assess differences in time to presentation to MDFT clinics over time. Models will be adjusted for age and sex. Where appropriate, interclass correlation (ICC) will be used to assess the proportion of the variation explained by referral to each different MDFT clinic. In case the ICC will be equal or greater than 10%, MDFT clinics will be included in the model as random effect. To model the impact of the programme since its implementation a before-and-after design will be employed. Specifically, baseline data collected in 2016, will be compared with data collected in 2019. Models will be adjusted for age and sex.

For a more robust evaluation of the programme the WSIC database will be used to select external controls, sampling from areas where the intervention has not been implemented. Doubly robust methods such as the inverse probability weighting regression adjustment will be employed to compare outcomes between attendees and non-attendees. Covariate selection to generate propensity scores will be based on a combination of what is observed empirically (e.g. covariates explaining differences between groups) and by what has been previously used in previous research. This approach would be appropriate to reduce selection bias associated with likelihood of attending the program associated with specific socio-demographic characteristics [20].

### Work Stream 2 – Patient and Staff Experience

A phenomenological, qualitative study to explore the narratives of people with diabetes and staff and their experience in the NWL Diabetes Foot Care Transformation project will be conducted. The success of this project relies on meeting the different challenges and capacities of both service users and providers. Service users might have different perceptions of what co-production, multi-disciplinary care and integrated care mean to them and these might be different to perception held by clinicians and academics. A whole-rounded inquiry into service user, provider, and commissioner experience will, therefore, be conducted. Semi-structured personal interviews alongside all stakeholder groups involved in the foot networks, including service users, primary care providers, commissioners and specialist foot teams will be conducted. By doing so the aim is to develop a deep understanding of the project with the view of providing suggestions for improvement. The authors have previously employed this approach in previous evaluations of integrated care initiatives) [21].

The inquiry into patient, provider and commissioner experience will focus on 5 areas of interest: 1. Exploring the concept of integrated care and meanings of multi-disciplinary and integrated care within the context of DFU. 2. Current challenges in service provision and how integrated multi-disciplinary care could help to alleviate these challenges. 3. Motivations to join the project, as a service user or a provider. 4. The value of co-production approaches. 5. Perception of the actual changes in care which have been happening during the implementation of the project and whether these changes provide the right response to their needs and expectations. There will be a focus on communication which is key to the success of multi-disciplinary care work. For services users this would focus on communication with providers, and for providers it will focus on communication with service users and other providers and commissioners.

#### Patient and Public Involvement

The participants will be participants of the NWL Diabetes Foot Network (service users, providers and commissioners) and people with diabetes attending MDFT clinics. The aim is to interview a sample of 7–10 people with diabetes, 7–10 providers and 7–10 commissioners in personal interviews, alongside one patient focus group, one provider focus group, one commissioner focus group and one mixed patient-provider-commissioner group. In earlier events arranged by the research team, it was found out that events where both staff and patients attended provided useful insights and reviews.

The interviews and focus groups will be audio-taped and transcribed verbatim while ensuring the interviewees’ anonymity. The interview will be designed to fit a period of 60 minutes. The protocol will be similar for the 3 groups, with adjustments to the dynamic of each interview or focus group. A coding process following by thematic content analysis will be carried out.

The qualitative inquiry will be complemented by a structured patient and staff survey, which will inquire into more general perceptions with a larger sample of participants. A survey to record patient experience, and a separate survey of provider experiences (NWL patient related experience measures survey; Appendix questionnaire 1) will be disseminated. Survey questions will explore similar issues to those in the qualitative strand, as detailed above. Outputs from the survey will be used to inform further interviews as required.

Ethics approval for this work will be sought. Before analysis, any identifying details will be removed from quotes. The survey would be anonymous, capturing non-identifying personal details.

### Work Stream 3 – Evaluation of the programme cost-effectiveness

The economic evaluation will include NHS and personal social services [22]. The analysis will be a within-trial cost-effectiveness analysis (pre and post 2017) for the NWL Diabetes Foot Care Transformation intervention against no intervention. The analysis will use resource data including: (a) development of training for health professionals; (b) support provided by podiatrists and specialists foot teams respectively; and (c) creation of digital foot care dashboard, and (d) and health and social service use.

Data will be collected through key informant interviews, and review of trial management records and the MDFT and WISC datasets. Unit costs will be taken from the standard unit costs (e.g. Personal Social Services Research Unit 2019) [23], and published literature. Costs that do not vary by use (e.g. development of digital foot care dashboard) will be costed separately and apportioned to participants appropriately. The main outcome of the economic analysis will be an incremental cost per change in the process indicators (e.g. number of referrals to MDFT clinics, number of amputations). Results will also be presented in the form of a cost-consequence analysis (disaggregated costs next to the important outcomes). Deterministic sensitivity analysis will explore; i) varying the mean cost of intervention based on health professionals’ input and ii) roll-out costs. Any subgroup analyses e.g. by medical condition, age group and gender will be exploratory.

Whilst this methodology is quite robust to assess the programme cost-effectiveness, a possible limitation will be the limited amount of data, reflecting that the programme has been implemented for less than three years. However, this approach will constitute an integrated part of the evaluation model which has to be considered as an ongoing process with updates in the evaluation given when longer follow-up data will be available.

### Dissemination

Findings from the different work stands will be discussed within the members of the Diabetes Foot Care Transformation Project and disseminated to patients and stakeholders through different partners including the NWL Diabetes Clinical Reference Group, the NWL Diabetes Foot Network, the NWL Clinical quality Leadership Group, the Imperial College Healthcare partners, and the NWL NIHR Applied Research Collaboration network.

## Discussion

This paper aims to describe the evaluation methodology for the NWL Diabetes Foot Care Transformation Project, a multidisciplinary and multifactorial programme aiming to improve health outcomes for individuals with diabetic foot complications needing care in NWL. Diabetes foot complications, such as DFU, place a huge burden on those affected in terms of quality of life and life expectancy and on health economies. Demonstrating the impact of multidisciplinary, integrated care models and the value of co-production approaches, such as in this transformation project, is important for health providers and commissioners trying to improve health outcome. Evaluation is also needed to identify strategies to overcome barriers which might have reduced the impact of the programme and key elements for improvement.

Diabetes and diabetes complications constitute a public health emergency not only in high income nations but also in lower- and middle-income countries. The World Health Organization recommends implementation of integrated care and multidisciplinary models and integration of these programmes across healthcare levels as a prerequisite of Universal Health Coverage. While fragmentation often characterise Health Systems in low- and middle-income countries (LMICs), several LMICs have attempted health system integration implementing such intervention [24]. Understanding how to implement such complex interventions in limited resource settings, where care pathways maybe very different is important. Another critical aspect to consider is the system ability to assess and evaluate the intervention, as data analysis and interpretation might depend on local business intelligence capacity.

Central to developing this evaluation methodology was to recognise the complex nature of the intervention [17]. Integrating care within the NWL diverse environment, would make attribution of cause and effect difficult. It is important to consider the tension between providing early evaluation results to inform decision makers against the need to undertake rigorous analytical methods. In this case, we multiple datasets will be analysed. Multi-disciplinary Diabetes Foot Team service data has been accurately recorded, as trained staff members continuously update it, but limitations associated with the use of this database have to be mentioned, including the lack of data on healed ulcers – one of the study outcomes – which has to be extracted from the National Diabetes Foot Audit, the lack of other clinical data (e.g. blood glucose), and the absence of patient information for the period before the enrolment into the programme. Data extraction from another external database, the WSIC, will be used to provide an external control that might improve causality. However, a longer follow-up period and a larger sample might still be needed to demonstrate change for all identified project goals. It should be considered that comparing improvement with other areas in the UK where innovation is being actively encouraged means that it is difficult to confirm if the control groups are genuinely intervention free. Furthermore, selection bias might arise when conducting service evaluation using real-world data as people who attended the service might differ in socio-economic characteristics from those who did not [25,26]. Therefore doubly robust methods will be used considering they have been shown to reduce this bias and avoid model miss-specification [27].

## Conclusion

The NWL Diabetes Foot Care Transformation Project, a multidisciplinary and multifactorial programme, was launched in 2017 in NWL to improve health outcomes for individuals with diabetic foot complications needing care in NWL. Evaluating this project will contribute to identify strategies to overcome barriers which might have reduced the impact of the programme as well as key elements for improvement. This evaluation is also important considering that diabetes and diabetes related complications constitute a public health emergency not only in developed nations but also in LMICs, where initiatives to promote Health System integration are being conducted. Understanding the true impact of such interventions in high income settings is important to consider translation and adaption in different settings where primary care might be quite different [28].

## Additional File

The additional file for this article can be found as follows:

10.5334/ijic.5956.s1 Appendix. Table 1, Figure 1 and Questionnaire 1.

## Disclaimer

This report is independent research supported by the National Institute for Health (NIHR) Research Applied Research Collaboration (ARC) Northwest London. The views expressed in this publication are those of the author(s) and not necessarily those of the NIHR or the Department of Health and Social Care.
