# Supplementary material for: Evaluation of the North West London Diabetes Foot Care Transformation Project: A Mixed-Methods Evaluation
Source: Int J Integr Care. 2022 Apr 5;22(2):4. doi: 10.5334/ijic.5956 (PMC8992770; doi:10.5334/ijic.5956)
Supplement: Appendix. — Table 1, Figure 1 and Questionnaire 1. [file ijic-22-2-5956-s1.pdf]

Appendix

Table 1. List of 8 North West London Clinical Commissioning Groups (CCG) as at November 2020

No.	CCG
1	Brent CCG
2	Central London CCG
3	Ealing CCG
4	Hammersmith & Fulham CCG
5	Harrow CCG
6	Hillingdon CCG
7	Hounslow CCG
8	West London CCG

Figure 1. Overview of NWL Diabetes Foot Network and relationship between the Foot Network and the Foot Project Group

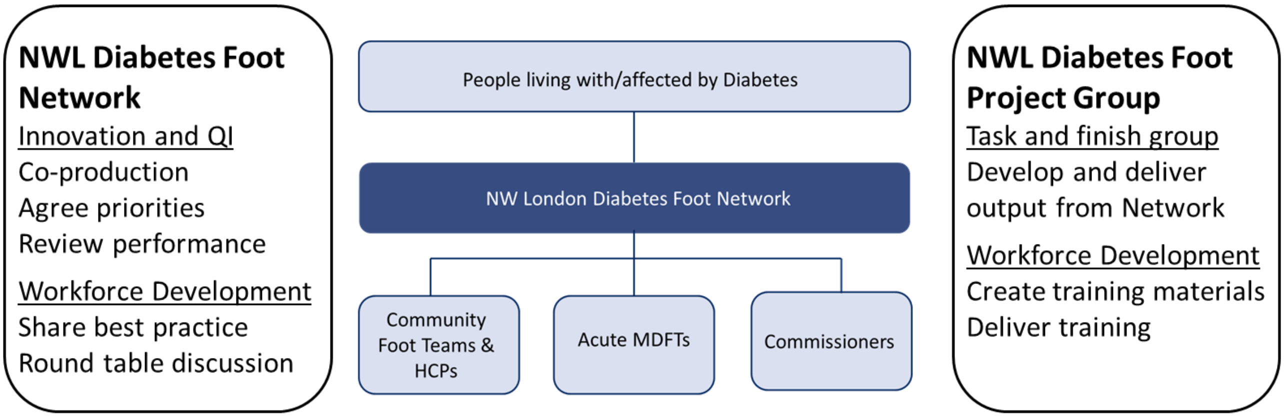

Questionnaire 1. NHS North West London Diabetes Transformation Network – Patient Reported Experience Measures (PREMs)

North West London Diabetes Transformation Network

PATIENT REPORTED EXPERIENCE MEASURES (PREMs)

Do you reside in NW London: ☐ Yes ☐ No

Which organisation(s) do your answers in this questionnaire relate to (please circle):

1. GP practice (please state which area) – Central London, West London, Hammersmith & Fulham, Hounslow, Ealing, Brent, Harrow, Hillingdon (need boxes for this)

2. Hospital (please state which one)…………………………………………………..........

3. Other (please state which service or NHS provider)…………………………………………………………………………….

1. **I was given information and support to help me manage my diabetes care (e.g the Knowdiabetes website) by my treating clinician.**					
Please tick the answer that best reflects how accurate you feel the above statement is.	Strongly disagree	Disagree	Neutral	Agree	Strongly Agree

2. **My emotional wellbeing was considered/asked about by my healthcare professional.**					
Please tick the answer that best reflects how accurate you feel the above statement is.	Strongly disagree	Disagree	Neutral	Agree	Strongly Agree

3. **I am satisfied with the support I received.**					
Please tick the answer that best reflects how accurate you feel the above statement is.	Strongly disagree	Disagree	Neutral	Agree	Strongly Agree

4. **I get timely access to care, appointments and information.**					
Please tick the answer that best reflects how accurate you feel the above statement is.	Strongly disagree	Disagree	Neutral	Agree	Strongly Agree

5. **The professionals involved in my care had adequate information about my diabetes.**

Please tick the answer that best reflects how accurate you feel the above statement is.	Strongly disagree	Disagree	Neutral	Agree	Strongly Agree
**6. I felt there was good communication between the different people/organisations involved in my diabetes care?**					
Please tick the answer that best reflects how accurate you feel the above statement is	Strongly disagree	Disagree	Neutral	Agree	Strongly Agree
**7. I feel my care was delivered in a safe way**					
Please tick the answer that best reflects how accurate you feel the above statement is.	Strongly disagree	Disagree	Neutral	Agree	Strongly Agree
**8. I am happy with the level of involvement I have in decisions about my diabetes care**					
Please tick the answer that best reflects how accurate you feel the above statement is.					
